# Low incidence of airborne SARS-CoV-2 in acute care hospital rooms with optimized ventilation

**DOI:** 10.1080/22221751.2020.1850184

**Published:** 2020-12-10

**Authors:** Nathan Dumont-Leblond, Marc Veillette, Samira Mubareka, Lily Yip, Yves Longtin, Philippe Jouvet, Bianka Paquet Bolduc, Stéphane Godbout, Gary Kobinger, Allison McGeer, Alex Mikszewski, Caroline Duchaine

**Affiliations:** a Centre de recherche de l’institut universitaire de cardiologie et de pneumologie de Québec, Quebec City, Canada; b Sunnybrook Research Institute, Department of Laboratory Medicine and Pathobiology, University of Toronto, Toronto, Canada; c Jewish General Hospital, Montreal, Canada; d Lady Davis Research Institute, Montreal, Canada; e Department of Pediatrics, Université de Montréal, St. Justine Hospital, Montreal, Canada; f Institut universitaire de cardiologie et de pneumologie de Québec, Québec, Canada; g Institut de Recherche & Development Agroenvironmental, Quebec City, Canada; h Département de microbiologie-infectiologie et d’immunologie, Université Laval, Quebec City, Canada; i Lunenfeld-Tanenbaum Research Institute, Sinai Health System, Toronto, ON, USA; j Department of Laboratory Medicine and Pathobiology, University of Toronto, Toronto, Canada; k The City University of New York, CIUS Building Performance Lab, New York, NY, USA; l Département de biochimie, de microbiologie et de bio-informatique, Faculté des sciences et de génie, Université Laval, Quebec City, Canada; m Canada Research Chair on Bioaerosols, Quebec City, Canada

**Keywords:** Bioaerosols, SARS-CoV-2, COVID-19, air, airborne exposure, healthcare settings

## Abstract

The worldwide repercussions of COVID-19 sparked important research efforts, yet the detailed contribution of aerosols in the transmission of SARS-CoV-2 has not been elucidated. In an attempt to quantify viral aerosols in the environment of infected patients, we collected 100 air samples in acute care hospital rooms hosting 22 patients over the course of nearly two months using three different air sampling protocols. Quantification by RT-qPCR (ORF1b) led to 11 positive samples from 6 patient rooms (*C_t_* < 40). Viral cultures were negative. No correlation was observed between particular symptoms, length of hospital stay, clinical parameters, and time since symptom onset and the detection of airborne viral RNA. Low detection rates in the hospital rooms may be attributable to the appropriate application of mitigation methods according to the risk control hierarchy, such as increased ventilation to 4.85 air changes per hour to create negative pressure rooms. Our work estimates the mean emission rate of patients and potential airborne concentration in the absence of ventilation. Additional research is needed understand aerosolization events occur, contributing factors, and how best to prevent them.

## Introduction

A novel Coronavirus (SARS-CoV-2) was discovered at the end of 2019, causing the Coronavirus disease 2019 (COVID-2019) [1]. The World Health Organisation (WHO) declared the COVID-19 pandemic on March 11, 2020 [2]. By the end of October 2020, nearly 43 million people worldwide were diagnosed with the disease and it had claimed more than 1.1 million lives [3]. The global repercussions of the pandemic have sparked important research efforts, including investigations into the transmission modes of this virus. Yet, there are still many gaps in the understanding of what may contribute specifically to virus transmission, and of how best to control its spread in the population and in healthcare settings.

The SARS-CoV-2 is a member of the *Betacoronavirus* genus, joining other known human pathogens, such as the SARS-CoV-1, responsible for the Severe Acute Respiratory Syndrome (SARS) 2003 epidemic [4]. Yu *et al.* generated a model of the residential buildings that were at the centre of this epidemic event and found strong evidence suggesting a role for airborne transmission (droplet nuclei) in the spread of SARS-CoV-1 [5]. The phylogenetic similarity of SARS-CoV-2 with SARS-CoV-1 led to concerns that it may be transmitted in a similar way. Van Doremalen et al. also evaluated the resistance to aerosolization and particles maturation in the air of those two viruses to be similar [6]. Moreover, infection events possibly associated with aerosol transmission have been reported in the United States, such as the Skagit Valley Choir incident where 45 persons were diagnosed with COVID-19 after a choir practice [7] (other routes of transmission were not ruled out). However, research teams who have attempted to detect viral particles in the air in healthcare settings using different sampling and detection methods have achieved mixed results with limited number of patients and replicates [8–11]. Guo et al. obtained positive samples in 35% (14/40) and 12,5% (2/16) of air samples taken in intensive care units and a general COVID ward, respectively, using a Wetted Wall Cyclone Sampler at 300L/min for 30 min and RT–PCR viral RNA detection (does not account for infectivity). Ong et al. could not detect SARS-CoV-2 in air samples using 37 mm cassettes with 0.3 µm polytetrafluoroethylene filters (4 h, 5 L/min) in the room of infected patients or with the Sartorius MD8 microbiological sampler (gelatin membrane filter, 15 min at 6m^3^/h) outside the room despite detecting the presence of the virus on inanimate surfaces (swabs). However, they found detectable virus by RT-qPCR on the air exhaust outlet that could be associated with aerosol deposition. Lui et al. also used gelatin filters and obtained null or very low concentrations of viral genomes per cubic metre of air (≤ 21 copies/m^3^). Binder *et al.* detected the virus by RT-qPCR in the room of only three of the twenty patients sampled using 8 NIOSH BC 251 samplers simultaneously. They could not cultivate the samples on Vero cells. Using the same samplers, Santapia *et al.* detected infective airborne SARS-CoV-2 particles using viral culture on Vero cells and microscopic observations, Wester blots in viral cultures and RT-qPCR analyses of cell culture [12]. The role of aerosols in SARS-CoV-2 transmission as yet to be established and needs to be further investigated. Clinical determinants of shedding and risk of aerosolization need to be identified and room ventilation parameters in clinical settings determined in order to estimate patients’ potential emission rates. A better understanding of the transmission route of SARS-CoV-2 will be a major asset in the development and application of mitigation and protection methods for healthcare workers and the general populations.

In this study, 22 acute care hospital rooms were sampled, housing COVID-19 patients that did not require intensive care, using multiple air samplers, sampling times and flow rates. The investigation of airborne viral load was performed and the different factors that can lead to increase aerosolization of the virus were assessed.

## Methods

### Hospital setting

This prospective observational study was conducted at the Institut Universitaire de Cardiologie et Pneumologie de Quebec (IUCPQ) between March 26 to June 6^,^ 2020. We sampled an adapted ward dedicated to patients with non-severe COVID-19 built in the 1950s (original air changes per hour of 0–0.1). Patients were not receiving critical care and were not intubated, but could be supplemented in oxygen via nasal cannula or on palliative treatment. The rooms sampled were transformed in negative pressure by replacing a window with a portative high flow air extraction device (average air volume of 48.5 m^3^). Based on measured flow rates of installed devices in relation to the volume of the room, the calculated mean air changes per hour was 4.85 (ranged from 3 to 7). Rooms were distributed on both sides of a central corridor and did not have an anteroom. The bathroom connection between rooms were sealed and patients used a commode chair that was emptied multiple times a day. The project was approved by the ethics committee of the Institut Universitaire de Cardiologie et de Pneumologie de Québec (IUCPQ) under the project identifier MEO-21-2021-3475.

### COVID-19 cohort

The air in the room of 22 patients was sampled. Convenience sampling was performed according to the patient availability. The detailed basic demographic and clinical data of patients are presented in Tables 1 and 2. The symptoms were recorded throughout the hospital stay of the patients and were not necessarily displayed on the day of sampling. Briefly, the average age of patients was 61.7 years and half (50%) were female; with a total length of hospital stay of 7.64 days. 86.36% of them survived. Eighteen participants experienced cough (81.82%) and sixteen had fever or dyspnoea (72.73%), twelve had both (54.54%). Nine patients had diarrhoea (40,91%), one had a sore throat (4.55%), seven presented headaches (31.82%), and six general fatigue and loss of appetite (27.27%). The majority of patients received non-invasive ventilation (57.14%) via nasal cannula of at least 1LPM of 100% O_2_ on the day of sampling. The others were not receiving oxygen on that day.

**Table 1.** Demographic and clinical data from the sampled cohort.

Patient ID	Age (years)	Sex	Total length of hospital stay (days)	Reported date of the first symptoms	Date of sampling	Number of days between the first symptoms and sampling	Supplementary oxygen (Nasal Canula)	Oxygen flow-rate (LPM)
A	75	F	13	2020-03-18	2020-03-31	13	+	5,6
B	74	M	11	2020-03-31	2020-04-09	9	+	2
C	54	M	4	2020-04-03	2020-04-14	11	-	
D	50	M	8	2020-04-14	2020-04-23	9	-	
E	38	M	15	2020-04-19	2020-04-30	11	+	2
F	94	M	2	2020-04-30	2020-05-05	5	+	N/D
G	72	F	8	2020-03-20	2020-03-26	6	+	>2
H	71	F	3	2020-03-14	2020-03-27	13	+	>2
I	51	F	4	2020-03-16	2020-03-31	15	+	>2
J	51	F	2	2020-03-23	2020-04-03	11	-	
K	84	F	8	2020-03-23	2020-04-07	15	+	3
L	79	F	2	2020-04-01	2020-04-07	6	+	0.5/2
M	55	F	2	2020-03-26	2020-04-08	13	-	
N	92	F	5	2020-03-25	2020-04-08	14	-	
O	76	M	6	2020-03-29	2020-04-09	11	-	
*P*	61	F	5	2020-04-04	2020-04-14	10	-	
Q	56	M	8	2020-04-01	2020-04-17	16	+	3
R	20	M	21	2020-03-26	2020-04-20	25	-	
S	52	M	18	2020-03-31	2020-04-22	22	-	
T	N/D	F	6	2020-04-21	2020-05-01	10	+	1
U	43	M	1	2020-04-27	2020-05-04	7	-	
V	N/D	M	16	2020-04-23	2020-05-01	8	+	N/D
Median	58.5		6			11		
Average	61.7		7.64			12

**Table 2.** Outcome and symptoms collected by the health professionals throughout the entire hospital stay.

Patient ID	Survival	Cough	Fever	Dyspnoea	Diarrhoea	Vomiting	Sore throat	Headache	Fatigue and loss of appetite	Myalgia
A	+	+	+	+	-	+	-	-	+	-
B	+	+	+	+	-	-	-	-	-	+
C	+	+	+	+	-	-	-	-	-	-
D	+	+	+	+	-	+	-	-	-	-
E	+	+	+	+	+	+	-	-	+	-
F	+	+	+	-	-	+	-	+	+	-
G	-	+	-	+	-	+	-	-	-	-
H	+	+	+	-	+	-	-	-	+	-
I	+	+	+	-	+	-	-	+	-	-
J	+	+	+	+	+	-	-	+	-	-
K	-	-	-	+	-	-	-	-	-	-
L	-	-	+	-	+	-	-	-	-	-
M	+	+	+	+	+	-	-	+	-	-
N	+	+	-	+	-	-	-	-	-	-
O	+	-	-	-	-	-	-	-	-	-
*P*	+	+	+	+	+	+	-	+	+	+
Q	+	+	+	+	-	-	-	-	-	-
R	+	+	+	+	+	-	-	+	+	-
S	+	+	+	+	-	-	-	-	-	-
T	+	-	-	-	-	-	-	-	-	-
U	+	+	-	+	+	-	+	+	-	+
V	+	+	+	+	-	+	-	-	-	-
Percentage of positives (%)	86.36	81.82	72.73	72.73	40.91	31.82	4.55	31.82	27.27	13.64

### Air sampling

A total of 100 samples were taken in the rooms of 22 patients over the course of their hospitalization (See the Supplementary Table 3 and Supplementary Figure 1 for the complete list and for room design). Therefore, the same environment could be sampled on multiple occasions during the patient stay. Patients who had short stay or were transferred to other facilities could not be resampled the following days. Air samples were taken using three different air samplers at multiple sampling times to optimize the capture of SARS-CoV-2 airborne particles. Filter elution volumes were also optimized.

Samples were collected simultaneously using two conductive plastic IOM (SKC, Eighty Four, PA, USA) with 3 µm gelatine filters (Sartorius Stedim Biotech, Gottingen, Germany) or one IOM and a 37 mm cassette with 0.8 µm polycarbonate filters (PC) (SKC, Eighty Four, PA, USA). Both were connected to the medical vacuum using a regulator (Genstar Technologies, Chino, USA) and the flow rates were adjusted to 10 L/min with the calibration adaptor (SKC, Eighty Four, PA, USA) using a portable flowmeter (TSI model 4199, Minnesota, USA). Sampling was performed for 4, 6 h, or 18 h simultaneously while the patient was in the room. The 4 and 6 h samples were taken during the day (between 9a.m. and 4p.m.) and the 18-hour samples overnight (between 4p.m. and 10a.m.). The IOMs and cassettes were hung from a foldable support approximately 1.5 m above the ground and placed at least 1.5 m bedside from the patient. They were located close to the wall behind the patients or behind the head of the patients to limit the collection of larger droplets (See Supplementary Figure 1). Of the 100 samples, 11 were taken with the SASS^®^ 3100 dry sampler at 300L/min for 15 min before installation or retrieval of the IOM and cassettes. The SASS^®^ was placed on the window sill at least 1.5 m from the patient.

### Sample Processing and storage

The samples were processed and stored on the day of their sampling. The RNA extractions and quantification were performed subsequently.

Gelatine filters were solubilized in 0.9 mL or 3 mL of viral transport media (VTM) (Redoxica, Little Rock, USA) in a 50 mL conical tube (Sarstedt, Newton, USA) and brought to 37°C until their dissolution. Due to difficulties obtaining VTM in the early weeks of the pandemic, samples up to 28 (Supplementary Table 3, Chronological Sampling Order) were solubilized in Dulbecco's Modified Eagle Medium (DMEM) + 10% foetal bovine serum (FBS) as described by Van Doremalen [6] . Both storage media were identified as equivalent in terms of cryoprotection and lack of interference with the RNA extraction process using RNA phages (data not shown). The resulting liquid was divided into 400 µL aliquots and kept at −80°C until further use.

The SASS® 3100 filters were eluted using the SASS®3010 particle extractor (Research International, Monroe, USA) with 5 mL of extraction buffer (138 mM sodium chloride, 2.7 mM potassium chloride, 0,05% Triton X-100, <0,1% sodium azide, 10 mM sodium phosphate). The eluate was then transferred to a concentration column (Amicon® Ultra-15, Merck Milipore Ltd., Tullagreen, Ireland) and centrifuged at 1000 g to a final volume of approximately 0.1 mL. The residual liquid was collected, and the column membrane washed with two rounds of 450 µL of VTM for a final volume of 1 mL. The column was briefly vortexed between each wash. The final 1 mL was divided into two 400 µL aliquots and kept at −80°C until further use.

The polycarbonate filters were eluted using 2 mL or 3 mL of VTM directly introduced in the 37 mm cassettes with a transfer pipet and ejected by the pressurizing action of a 50 mL empty syringe at the opposite opening of the cassette. The residual liquid was collected in a 50 mL conical tube, was divided into 400 µL aliquots, and kept at −80°C until further use.

### RNA extraction

RNA extracts were prepared from 400 ul of each sample using the MagMAX™ Viral RNA Isolation Kit (Applied Biosystems, Vilnius, Lithuania), according to the manufacturer's instructions. The beads were eluted with 50 µL of elution buffer and stored at −80°C until quantification. An extraction blank (no template control) was performed for each batch of samples treated.

### Quantification

The RT-qPCR reactions were performed using the Bio-Rad iTaq Universal Probes One-Step kit (California, USA). Briefly, 20 µL reactions were performed using 10 µL of iTaQ mix, 0.5 µL of iScript reverse transcriptase, 0.1 µL of 100 uM forward and reverse probe (final concentration = 0.5 uM/L), 0.05 µL of 100 µM probe (final concentration = 0,25 µM/L), 4.35 µL of molecular grade water, and 5 µL of the sample. The primer and probe sequences were designed by Chu et al. and are available in Supplementary Table 1 [13]. The detection was performed in duplicates for both target (ORF1b and N) for each sample. No template and extraction controls were tested for each batch. Plasmid positive controls were included with each batch to allow detection and quantification (2019-nCoV_N positive control plasmids from IDT [14] and a custom plasmid with the ORF1b insert) (Supplementary Table 1). Quantification was achieved based on ORF1b plasmid standard curve with a limit of detection of 1 plasmid/qPCR reaction. The amplicons were kept at −80°C and single positive (for either N or ORF1b) were sent to the sequencing platform to confirm specificity of the qPCR reaction. The estimation of the viral load in samples was achieved by mathematical transformations of the ORF1b plasmid standard curve for each RT-qPCR (see Supplementary materials). Mean emission rates for patients with positive air samples were calculated based on the viral load estimated by RT-qPCR ORF1b, volume of patient room, and averaged ACPH (Formula 1). These calculations assume a homogenous distribution of viral particles in the air of the patients’ room at steady state.

$$\text{Emission rate}=C_{v}\times V_{R}\times \text{ACPH}$$

where *C_v_* = Virus concentration per volume of air (genomes/m^3^)

*V_R_* = Total volume of the room (m^3^);

ACPH = Air exchange rate (number of changes/h)

### Sequencing

The RT-qPCR amplicons of samples that were only positive for one target were sent for Sanger sequencing to the Centre de Recherche du CHU de Québec (Québec, Canada), using the forward primers described in Supplementary Table 1.

### Viral culture

Vero E6 cells (African green monkey cells; ATCC) were maintained in DMEM (Wisent) supplemented with 10% FBS (Wisent), 2 mM L-glutamine (Wisent), and 100 U/mL penicillin and 100 μg/mL streptomycin (P/S, Wisent). Samples with a higher concentration of SARS-CoV-2 estimated by qPCR were attempted for culture (samples 4,5,7,8,10, and 11; Table 3). Vero E6 cells were seeded at a concentration of 3×10^5^ cells/well in a 6-well plate. The next day, 400 μl of PCR-positive sample was spun at 10,000 rpm to remove debris and the supernatant supplemented with 16 μg/mL TPCK-treated trypsin (New England Biolabs) and 2X antibiotic-antimycotic (Wisent) was added to the cells. The plate was incubated for 1hr in a humidified 37°C incubator with 5% CO_2_ and rocked every 15 min. After 1hr, the inoculum was removed and replaced with 2 mL of DMEM supplemented with 2% FBS, 2X antibiotic-antimycotic, 100 U/mL/100 μg/mL P/S and 6 μg/mL TPCK-treated trypsin. The plate was returned to the incubator and observed for cytopathic effect (CPE) for 5 days. If no CPE was observed, the supernatant (500ul) was passaged onto fresh Vero E6 cells and observed for another 5 days.

**Table 3.** Positives air sample results.

Sample ID	Patient ID	Day	Air Sampler	Sampling time (hours)	N target	ORF1b target	
					Ct	Ct	Viral concentration (genomes/m^3^)
1	A	1	IOM	6	39.05	-	-
2	B	1	IOM	6	39.5	33.46	208.33
3	C	1	IOM	6	39.8	-	-
4	D	1	IOM	6	36.75	35.15	63.79
5	D	1	37 mm cassette	6	37.45	33.01	335.42
6	E	1	37 mm cassette	6	39.31	-	-
7	F	1 (night)	IOM	18	36.46	32.31	187.5
8	F	1 (night)	37 mm cassette	18	35.48	32.07	514.17
9	F	1 (day)	37 mm cassette	6	38.74	35.14	9.86
10	F	2 (night)	IOM	18	38.01	34.93	23.25
11	F	2 (night)	37 mm cassette	18	37.36	33.46	270.83

## Results

100 air samples were collected (see Supplementary Table 3). Only 11 (from 6 different patients) were considered positive (Ct under 40), 3 of which were positive for the N RT-qPCR target (Ct = 39.05,39.31,30.8) but not ORF1b. The quantification results, as well as the clinical data of the corresponding patient, can be found in Tables 1–3.

Of the 11 patients (11/22, 50%) that were sampled over multiple days, only two had positive air samples. The room of patient A was sampled over four consecutive days. Only one positive sample was recovered from one of the two samplers (IOM) used on the second day (Ct N = 39.05). However, the room of the patient F repeatedly tested positive for both targets over the course of two days of consecutive sampling (night and day) (Table 3). The rooms of patients B,C,D, and E were sampled only once and lead to positive results for at least one of the two samplers and RT-qPCR targets. In those, the room of patient D was the only one that turned out to be positive for both sampler (IOM and cassette).

Clinical Ct values of screening and follow-up nasopharyngeal swabs are available in Supplementary Table 2. Only partial data is available as details of tests could not be retraced for every patients. Patients B et C were the only ones with positive air samples for whom we have the Ct values. Patient B was tested one day after his room was sampled with a *C_t_* value of 31. On the other hand, patient C tested negative the day after sampling. Patients without positive air samples (P,Q,R,S,V) had either a positive test with high Ct values (29.4 or 34.5/37.2), a negative test or no test close to the date of sampling (V).

Only 3 patient's room samples (B, D and F) led to an estimation of the SARS-CoV-2 load in air. For each of these samples, emission rates were calculated using measured SARS-CoV-2 per cubic meter, sampling time, room volume and ACPH. When multiple samples were available, emission rates were averaged. For patients B, D and F, estimated mean emission rates were respectively 5.2E+04, 4.76E+04 and 4.71E+04 virus genomes per hour for on overall average emission and concentration of 4.86E+4 virus genomes per hour.

The 6 patients in rooms in which SARS-CoV-2 was detected in the air were 64.17 years old on average, predominantly male (5/6, 83%) and had a slightly longer total hospitalization than the average of the whole cohort (9 days vs. 7.64 days). Contrary to the cohort, every patient experienced fever (100% vs. 72.73%), dyspnoea (100% vs. 72.73%) and cough (100% vs. 77.27%). Only patient E experienced diarrhoea (16.67% vs. 40.91%). Four of the six patients with positive air samples were receiving non-invasive ventilation treatment via nasal cannula. The 6 patients happened to be placed in six different rooms throughout our COVID ward.

Even when the IOM and cassette were positive (patient D), SARS-CoV-2 could not be recovered in the air using the SASS®3100 according to our experimental protocol. The majority of positive air samples were collected using IOMs (7/11, 63.64%). When both IOMs and cassettes were used (patients D, E, and F), cassettes lead to positive results more often than IOMs (5/5 vs. 3/5) and higher concentrations overall (Table 3).

## Discussion

On the 100 samples taken, only 11 were positive. These results are in line with the findings of other research groups [8–11], who also noted negative or low concentrations of viral RNA in many of their samples. Therefore, virus-laden bioaerosols may be produced in low quantity by patients or are rapidly removed from the environment through air-handling systems. On the other hand, the additional physical stress applied to viral particles during sampling and filter elution could have led to the degradation of the genomic material and the underestimation of viral content in air, as well as reduction of viability. However, a higher concentration of viral RNA (from 2 to 3 logs) was detected in the air samples compared to a related study that attempted quantification by Lui *et al.* [8]. The divergence in sampling flow rates (5 L/min vs. 10 L/min), sampling time (4,6,18 h vs. 7 days), extraction technique (TRIzol LS vs. MagMAX™ Viral RNA Isolation Kit) and detection method (RT-ddPCR vs RT-qPCR) may explain these differences. Mostly, the lower sampling flow rates over a much longer period of time might have allowed them to collect multiple aerosolization events throughout the week, but might have led to a greater degradation of the viral particles of the filter.

On the contrary, airborne viral concentrations obtained in this study are lower than those reported by Chia *et al.* using NIOSH BC 251 samplers [15]. Adding the concentrations of viral particles they detected in aerosols larger than 4 μm to those between 1 and 4 μm, total concentrations of 1.84 × 10^3^–3.38 × 10^3^ RNA copies/m^3^ were measured. However, elution liquids from six NIOSH samplers ran at once in each room were pooled. Here, only two samplers were run at once and their filter were treated separately. Even if the total volume of air sampled is similar, due to different sampling rate (3.5L/min vs 10L/min), the presence of multiples air intakes (6 samplers vs 2 samplers) may have increased the chances of particle capture. In addition, the air samplers were placed closer to the patients (1 m vs 1.5 m) which may have increased the likelihood of collecting larger particles (>4 μm). Their air exchange rates were also higher (12 per hour) and other clinical variable, such as clinical Ct values, could weight in these variations.

### Concentration, emission rates, and air exchange rates

All data were collected in rooms with moderate air exchange rates. Rooms ventilation may mask the actual bioaerosols production by a given source. Measured emission rates from 3 patients led to similar results with a mean emission of 4.86E+4 (ranging from 4.71E+04–5.2E+04) SARS-CoV-2 genomes. Given these results and the fact that all sampled rooms in this study were similar in volume and air exchange rates, these emission rates from patients probably represent the limit of detection of the actual protocol.

In the present study, the rooms were in negative pressure using portative high flow air extraction devices. Knowing that ventilation contributes to air renewal and reduces the chances of observing aerosol buildup, lower ventilated rooms would possibly lead to higher concentration and ultimately workers exposure. In fact, many hospitals use a passive air exchange allowing a very low air change estimated at 0,5 ACPH (2 h for a full-room air change). In that case, the concentration in the room could be much higher than the sample study rooms. Assuming a same average emission rate, the concentration in the room could reach 2004.1 genomes/m^3^ after two hours instead of the 201,6 genome/m^3^ measured in the present study underlining the importance to adopt adequate ventilation and air exchange.

### Origin of contamination and Influencing factors

The inconsistency of the findings of this study seems to indicate that the presence of SARS-CoV-2 in the air is more complex and multifactorial than it may seem and require case-by-case risk evaluation. Therefore, the severity of symptoms to be an indicator of viral shedding has been investigated using observations of patients’ symptoms. All the rooms in which positive air samples were found housed patients presenting coughing, fever, and dyspnoea. It represents a slightly higher proportion than the overall cohort. As the action of coughing is known to produce a significant amount of airborne particles [16], the development of this symptom could lead to high concentration of airborne viral particles in the environment of patients. However, the constant evolution of the sickness and possible variability in symptoms monitoring does not allow us to conclude a strong association between the severity of those symptoms and the presence of the virus in the air. In addition, a partial dataset of clinical Ct values can be found in Supplementary Table 2. They are measurements of the viral load in the nasopharynx of patients at multiple moments during their stay. Ct values do not seem to be a predictor of the presence of the virus in the air. Considering the large number of missing values, strong conclusions related to clinical Ct values cannot be drawn.

The length of hospital stay could also be used as an indicator of symptom severity. The average length of stay for the patient in positive rooms was close to the overall cohort (9 vs. 7.94 days). It does not seem to be an appropriate indicator of aerosolization risks. On the other hand, oxygen supplementation is known to pose a risk of particles aerosolization [17]. The patients with positive air samples did not all receive supplementary oxygen, neither did they receive it in a greater proportion than the patients without positive air samples (66,67% vs. 60%). We found no clear correlations between the clinical data available to us and the presence of SARS-CoV-2 in the air of patients’ room. However, the relatively low number of patients in this study does not allow to confirm an absolute lack of correlation between airborne viral shedding and clinical variables.

Because of varying symptomatic levels and total viral load in the organism [18], the stage of infection or time since the development of the first symptoms could be an important factor in airborne viral shedding. In an attempt to catch the prime shedding period, air was sampled in 11 patients’ rooms over multiple days. Viruses were detected in the air of the patient F repeatedly (thrice) for a total period of 60 h. Even if normalized by the total volume of air sampled, 18 h samples allowed the detection of a higher concentration of viral genomes than the 6 h samples (23,25 - 514,17 genomes/m^3^ vs. 9,86 genomes/m^3^). Therefore, multiple aerosolization events may have occurred and the viral particles released in the air could have been captured many times and additively during those 18 h. On the other hand, our inability to detect aerosolized viruses over four consecutive days in the room of patient A might be associated to lower particles production overall, since we detected a fairly low amount of viral genome on day 2 (Ct N = 39.05). We were unable to detect SARS-CoV-2 in the rooms of the 9 other patients that were sampled over multiple days.

The cohort had been sick for an average of 12 days before our first sampling with a median of 11 days (Table 1). The positive patients do not seem to deviate from that distribution. The number of days since the development of the first symptoms, or the stage of infection, does not seem to indicate the presence of viral RNA in the air. However, patients A to F had been sick for 5–13 days before their room was sampled. The viral RNA detected could be the product of viral shedding in the air long after the first appearance of symptoms, even up to two weeks, or just the re-aerosolization of RNA molecules emitted previously. Our inability to cultivate any of our samples seems to indicate that these viral particles were not infectious. These results support other findings regarding the extended period in which viral RNA can be detected in patients’ faeces (up to 33 after a negative nasopharyngeal swab) [19], saliva [20], and even nasopharyngeal swab after the resolution of symptoms [21].

No live virus was isolated from air samples, either due to viral inactivation through the sampling process or the true absence of whole, infectious virions. In the absence of supporting experimental data with SARS-CoV-2, the sensitivity of virus isolation for viral bioaerosols is relatively unknown. The use of condensation-based air samplers may improve infectivity conservation [22]. However, a recent study suggests the use of electronic microscopy and viral protein quantification on cultivated and infected cells as an appropriate indicator of viral infectivity. It would allow detection when other techniques based on macroscopic observation (plaques) may fail [12]. Median dose–response for SARS-CoV through nasal instillation was proposed to be 280 PFU. Such data is not available for COVID-19 virus [23]. Nonetheless, given the low concentrations of viral RNA recovered from air, the likelihood that aerosols contained sufficient quantities of inhalable infectious virions to cause transmission appears low.

### Methodological challenges

As for most situations, no single air sampler can be identified as greatly superior or solely appropriate to sample airborne viruses in healthcare settings [24]. Hence three different samplers were tested to increase chances of finding viral particles if present: IOMs, 37 mm PC filter cassettes and SASS®3100. The initial methodology was based on the work of Lui et al. who used gelatin filters to recover SARS-CoV-2 as they were one of the first to report positive air samples for the virus [8]. However, a different filter support was chosen (IOMs) because of its broad range of particle collection (cutoff at 100 µm), good capture efficiency under 10.6 L/min [25], and our previous successful uses in other contexts [26]. The undiscriminating nature of this sampler in terms of particle size was beneficial as it was also able to capture the breathable particles that may be of interest in cases of respiratory disease transmission. However, this nonspecificity also restricts possibility of identifying which type of particles were captured. From the 23rd sampling going forward (See Supplementary Table 3, Chronological sampling order), 37 mm cassette with PC filters were included in combination with an IOM, to compare the efficiency of the two devices.

The low number of positive samples does not allow to form strong conclusion regarding the efficiency of samplers but, in our experimental conditions, the 37 mm cassettes with PC filters seem to have outperformed the IOMs coupled with gelatine filters. When used in parallel, cassettes led to a higher rate of positive then IOMs (5/5 vs. 3/5) and could detect SARS-CoV-2 on two instances where IOMs could not. Burton *et al.* observed a better physical collection efficiency (not taking into account viability) of biological particles by gelatine filter versus PC filters both in 37 mm cassettes [27]. Therefore, we hypothesize that the sampler design might have a significant role in particle capture and the preservation of the viral genome integrity. The narrower opening of the 37 mm cassettes compared to IOMs may accelerate particles more efficiently for a same flow rate and cause impaction on our filter. The elution of the filters (dissolution vs. rince) may also play a role in the recovery of viruses and their integrity by avoiding supplementary stress, e.g. heating.

In an attempt to capture more viruses, the volume of air sampled was increased using a high-flow electret filter sampler. However, the protocol put in place using the SASS® 3100 was unsuccessful at collecting and detecting the viruses, even when other samplers allowed collection on the same day of sampling. The short sampling time of the instrument (15 min) could have reduced chances to catch aerosols that are occasionally and randomly produced by the infected patient. The filter elution or concentration techniques could also have been detrimental to the viral integrity, leading to RNA degradation and loss of signal, due to the mechanical stress applied by the SASS® extractor (high-frequency vibrations), the presence of detergent in the filter extraction solution (Triton X-100), and vortexing or centrifugation during concentration. In addition, the high flow rate (300L/min) compared to the other samplers may have increased viral desiccation and degradation. The placement of the sampler, possibly outside the airflow stream, may have reduced our capacity to detect the virus. Due to small sample size, these results alone do not prove that this sampler is inappropriate for the study of airborne SARS-CoV-2, but it may still be an indicator of its relatively low efficiency when used according to our protocol.

### Potential airborne transmission

In the sampled environment (optimized air exchange rates, trained personnel, increased cleaning of surfaces, cohorting of positive patients and dedicated personnel), between March and June, low nosocomial infection was reported, both for healthcare professionals (only one) and patients (none). It was not the case for many health care settings around the world. Even if many parameters can influence the efficiency of nosocomial transmission prevention, such as appropriate PPE and infection control practices, consideration for optimal ventilation may have a central place in the effective contamination containment strategies to prevent and control nosocomial dissemination. In fact, mathematical models involving various scenarios of contact between infected individuals and recipient propose that ventilation rates have a direct impact on the maximum exposure contact time to reach acceptable maximum individual risk of infection [28]. However, personal protective equipment impact on exposure should supplement this model to better estimate the real occupational risk. Data provided in this article cannot assess with accuracy the importance of ventilation on infection prevention. Yet, it seems reasonable to assume that it could reduce viral accumulation in the environment of patients and reduce the potential risk of airborne exposure. Additional research is needed to establish quantitatively the influence of ventilation on airborne virus transmission.

Since the virus was detected in the air on some occasions, and recurrently for one patient, the risk of airborne transmission still has to be considered and further work needs to investigate what may favour the aerosolization of this virus and determine its ability to survive in the air and settle on surfaces. The emission dynamics are not characterized in our report and, although mean emission rates were estimated, they could come from a wide variety of emission situations, from rare events to more constant emission.

## Conclusion

In the studied healthcare environment, the airborne viral loads of aerosols are anecdotal in most scenarios, and are not predicted by symptomatology, supplementation of oxygen or other clinical variables. Our results suggest that in acute hospital settings, properly ventilated rooms may not allow significant accumulation of bioaerosols. However, impact of sampling process on viral integrity is not understood and underestimation of the viral load may be happening. In this study, there was no procedure leading to high risk of aerosol generation (intubation, manual ventilation). Evaluation of aerosol generating procedures in intensive care is to be performed to fully understand and describe if those situations lead to increased exposure risks for workers.

## Supplementary Material

Clean_copy_of_supplementary_material.docx

Supplementary_Table_3.xlsx
