# Supplementary material for: Low incidence of airborne SARS-CoV-2 in acute care hospital rooms with optimized ventilation
Source: Emerg Microbes Infect. 2020 Dec 10;9(1):2597–605. doi: 10.1080/22221751.2020.1850184 (PMC7734095; doi:10.1080/22221751.2020.1850184)
Supplement: Clean_copy_of_supplementary_material.docx [file TEMI_A_1850184_SM9684.docx]

Supplementary materials for the article:

**Low incidence of airborne SARS-CoV-2 in acute care hospital rooms with optimized ventilation**

Nathan Dumont-Leblond^1^, Marc Veillette^1^, Samira Mubareka^2^, Lily Yip^2^, Yves Longtin^3,4^, Philippe Jouvet^5^, Bianka Paquet Bolduc^6^, Stéphane Godbout^7^, Gary Kobinger^8^, Allison McGeer^9,10^, Alex Mikszewski^11^ and Caroline Duchaine^1,12,13^

1. Centre de recherche de l’institut universitaire de cardiologie et de pneumologie de Québec, Quebec City (QC), Canada

2. Sunnybrook Research Institute and Department of Laboratory Medicine and Pathobiology, University of Toronto, Canada

3. Jewish General Hospital, Montreal, Canada

4. Lady Davis Research Institute, Montreal, Canada

5. Université de Montréal, St. Justine Hospital, Department of Pediatrics, Montreal, Canada

6. Institut universitaire de cardiologie et de pneumologie de Québec, Québec, Canada

7. Institut de Recherche & Development Agroenvironmental, Quebec City, Canada

8. Département de microbiologie-infectiologie et d’immunologie, Université Laval, Quebec City, Canada

9. Lunenfeld-Tanenbaum Research Institute, Sinai Health System, Toronto, Ontario

10. Department of Laboratory Medicine and Pathobiology, University of Toronto, Toronto, Canada

11. The City University of New York, CIUS Building Performance Lab, New York, New York, USA

12. Département de biochimie, de microbiologie et de bio-informatique, Faculté des sciences et de génie, Université Laval, Quebec City, Canada

13. Canada Research Chair on Bioaerosols

*Corresponding author. E-mail : [Caroline.Duchaine@bcm.ulaval.ca](mailto:Caroline.Duchaine@bcm.ulaval.ca)

This document includes:

Supplementary Figures 1

Mathematical transformation of data

Supplementary Tables 1 to 2

**Supplementary Table 1 : SARS-CoV-2 qPCR primers, probes and plasmid insert (1)**

| Identification | Sequence |
| --- | --- |
| ORF1b Forward | 5’-TGGGGYTTTACRGG TAACCT-3’ (Y ¼ C/T, R ¼ A/G) |
| ORF1b Reverse | 5’-AACR CGCTTAACAAAGCACTC-3’ (R ¼ A/G) |
| ORF1b Probe | 5’-TAGTTGTGATGCWATCATGACTAG-3’ (5’-FAM/ZEN/3’-IBFQ format; W ¼ A/T) |
| ORF1b control | TTCAACAATGGGGTTTTACAGGTAACCTAC AAAGCAACCATGATCTGTATTGTCAAGTCCATGGTAATGCACATGTAGCTAGTTGTGATGCAATCATGACTAGGTGTCTAGCTGTCCACGAGTGCTTTGTTAAGCGTGTTGACTGGACTATTG |
| N Forward | 5’ -TAATCAGACAAGGAACTGATTA-3’ |
| N Reverse | 5’ -CGAAGGTGTGACTTCCATG-3’ |
| N Probe | 5’ -GCAAATTGTGCAATTTGCGG-3’ (5’-FAM/ZEN/3’-IBFQ format) |

Reference :

1. Chu DKW, Pan Y, Cheng SMS, Hui KPY, Krishnan P, Liu Y, et al. Molecular Diagnosis of a Novel Coronavirus (2019-nCoV) Causing an Outbreak of Pneumonia. Clin Chem [Internet]. 2020 Apr 1 [cited 2020 Jun 17];66(4):549–55. Available from: https://www.gisaid.org/;

Mathematical transformation of data

Legend:

$$V_{air}=Volume of air per volume of elution liquid$$

$$P=Proportion of sample quantified$$

$$V_{equivalent}=Volume of air per RT-qPCR reaction$$

$$C =Concentration of genome per volume of air$$

$$V_{air}=\frac{Sampling flow rate \left( \frac{L}{min} \right)\times Sampling duration (min)}{Elution volume (mL)}$$

$$P= \frac{Volume of liquid extracted (\mu L)}{Final extraction volume (\mu L)} \times Volume of extract per reaction (mL)$$

$$V_{equivalent}=V_{air} \times P$$

$$C=\frac{Number of genomes}{V_{equivalent}} \times\frac{1000L}{{1 m}^{3}}$$

Exemple:

For a sample with 300 genomes copies taken with an IOM for 6 hours

$$V_{air}=\frac{10 \left( \frac{L}{min} \right)\times360 (min)}{0.900 (mL)}$$

$$P= \frac{400 (\mu L)}{50 (\mu L)} \times0.005 (mL)$$

$$V_{equivalent}=4000\frac{L}{mL}\times0.04 mL$$

$$C=\frac{300copies}{160L} \times\frac{1000L}{{1 m}^{3}}$$

$$C=1875 copies/m^{3}$$

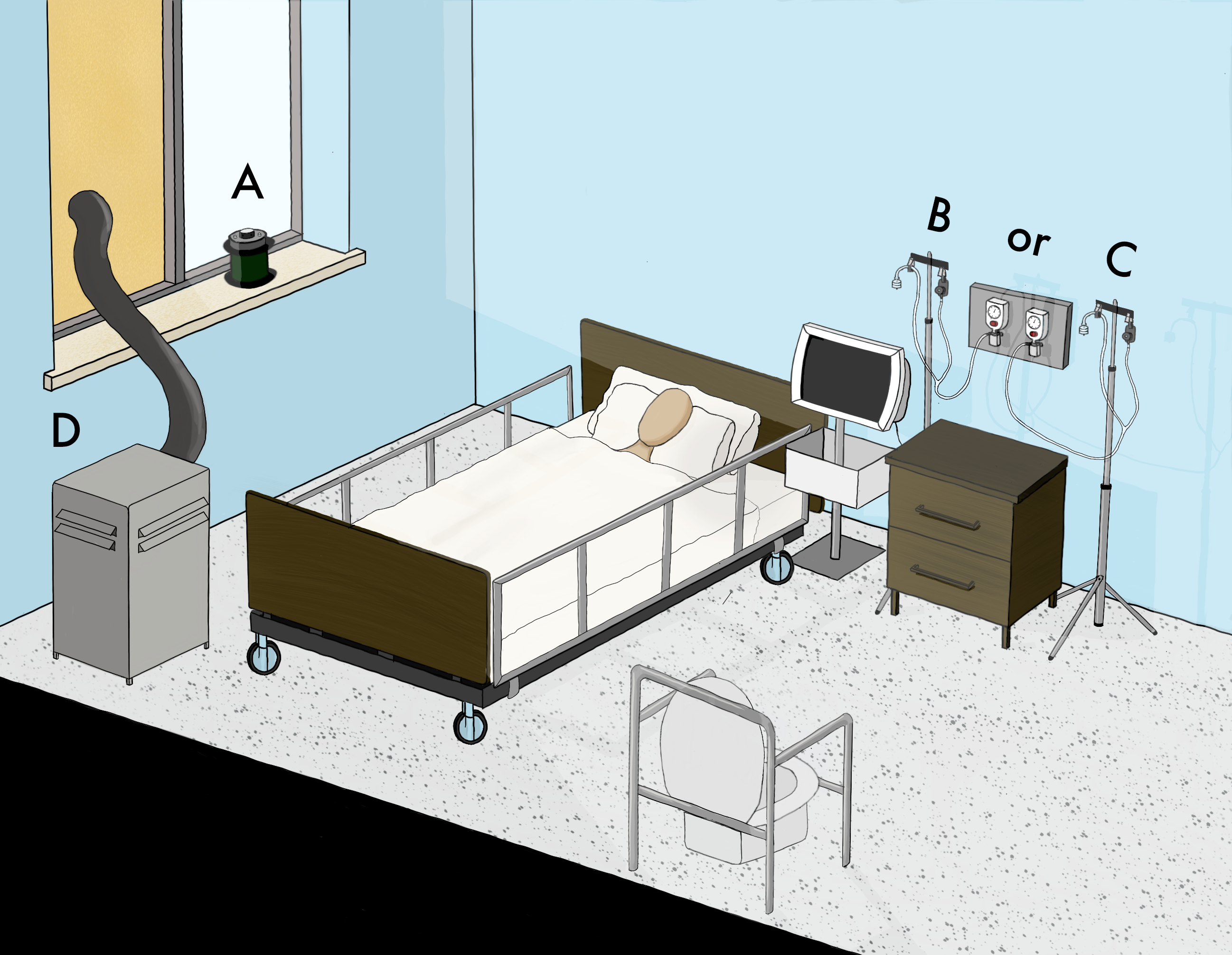


**Supplementary Figure 1: Schematic representation of the sampled hospital rooms. The SASS 3100® was placed on the window sill (A). The 37mm cassette and IOM were placed behind the patients’ head, either at position B or C according to the space available. The portable air extraction device was put at the feet of the patients (D).**

| Patient ID | Date of  screening | Date of the first  air sampling | Nasopharyngeal test results* | Ct value* | Presence of viruses in the air of the room |
| --- | --- | --- | --- | --- | --- |
| B | 04/10/2020 | 04/09/2020 | + | 31 | + |
|  | 04/14/2020 |  | - |  |  |
|  | 04/15/2020 |  | - |  |  |
| C | 04/15/2020 | 04/14/2020 | - |  | + |
| P | 04/14/2020 | 04/14/2020 | + | 29.4 | - |
|  | 04/18/2020 |  | + | 31.3 |  |
| Q | 04/15/2020 | 04/17/2020 | + | 34.5 | - |
|  | 04/19/2020 |  | + | 37.2 |  |
| R | 04/15/2020 | 04/20/2020 | + | 27.8 | - |
|  | 04/16/2020 |  | + | 32 |  |
|  | 04/20/2020 |  | - |  |  |
|  | 04/21/2020 |  | - |  |  |
| S | 04/15/2020 | 04/22/2020 | + | 27.7 | - |
|  | 04/16/2020 |  | + | 32.1 |  |
|  | 04/21/2020 |  | - |  |  |
|  | 04/22/2020 |  | - |  |  |
| V | 04/25/2020 | 05/01/2020 | + | 24.8 | - |
|  | 05/06/2020 |  | - |  |  |
|  | 05/07/2020 |  | - |  |  |

**Supplementary Table 2 : Quantification results of SARS-CoV-2 screening nasopharyngeal swabs from enrolled patients**

*Tests were performed on the BD Max™ plateforme (Becton-Dickson, USA). RNA extraction reagents : ExK™ TNA-3 (Becton-Dickson, USA), RT-qPCR reagents: RIDA^®^ SARS-CoV-2 RUO (R-Biopharm, Germany).
